# Psoriasiform Drug Eruption following Fremanezumab: A Case Report

**DOI:** 10.1159/cde/abvag005

**Published:** 2026-08-26

**Authors:** Kayla Coffman, Madelyn Ross, Emily Saurborn, Ashley Grimmett, Shane Cook

**Affiliations:** Department of Dermatology, Joan C. Edwards School of Medicine, Marshall University, Huntington, WV, USA

**Keywords:** Psoriasiform drug eruption, Inflammation, Calcitonin gene-related peptide inhibitors, Case report

## Abstract

**Introduction:**

Calcitonin gene-related peptide (CGRP) inhibitors such as fremanezumab are used for migraine prevention. Although relatively safe, adverse skin reactions after beginning CGRP inhibitors have been reported in the literature. We present a case report on a patient who presented with a psoriasiform drug eruption 2 weeks after beginning fremanezumab.

**Case Presentation:**

A 32-year-old female with no prior history of psoriasis had a psoriasiform drug eruption that began 2 weeks after injection of the CGRP inhibitor fremanezumab. The eruption began localized to the injection site and later spread to involve the trunk and extremities. Skin biopsy revealed histologic findings consistent with psoriasiform features. Discontinuation of the CGRP inhibitor led to the resolution of all skin manifestations.

**Conclusion:**

The complex effect of CGRP inhibitors on immune modulation may increase the risk of inflammatory skin reactions. These skin manifestations are important for clinicians to be aware of in patients with and without a history of inflammatory conditions. This case report offers further insight into the possible immune system effects present in inflammatory conditions such as psoriasis.

## Introduction

Fremanezumab is a monoclonal antibody targeting calcitonin gene-related peptide (CGRP). It is an effective option for migraine prevention and is becoming widely used.

CGRP inhibitors were first approved by the Food and Drug Administration in 2018 for migraine prevention. A randomized, double-blind, placebo-controlled, parallel-group trial conducted in 2018 reported the benefits of fremanezumab and noted the most common adverse events were injection site pain, induration, and erythema. At that time, no immunological or dermatological reactions to the drug were reported [1]. However, post-marketing evidence has revealed potential for inflammatory reactions. A case series in 2021 reported eight patients with inflammatory reactions following the initiation of CGRP inhibitors. Six of the eight cases developed new inflammatory conditions post-exposure, while the other two patients had an exacerbation of their previously well-controlled immunological diseases [2]. These previous case reports highlight the potential for CGRP inhibitors not only to cause dermatological reactions but also inflammatory manifestations.

This case report highlights a potential association between a CGRP inhibitor and a psoriasiform drug eruption. Clinicians should be aware of the potential for immunological presentations in susceptible patients taking CGRP inhibitors along with the importance of skin biopsy in the diagnosis of refractory skin lesions. The CARE reporting guidelines were followed in preparation of this manuscript. The completed CARE checklist is available as online supplementary material (for all online suppl. material, see https://doi.org/10.1159/cde/abvag005).

## Case Presentation

A 32-year-old female with a past medical history significant for chronic migraine presented with a rash involving the trunk and extremities that developed after beginning Fremanezumab-vrfm 225 mg/1.5 mL subcutaneous auto-injector every 30 days for migraine prophylaxis. The patient presented to an internal medicine walk-in clinic with a pruritic skin eruption that began approximately 2 weeks after starting the medication, initially localized to the injection site in the abdomen, then progressed to involve the trunk and extremities. She had begun a 6-day prescription of tapered 4 mg oral methylprednisolone that offered minimal relief. Patient denied any new exposures to detergents, soaps, or foods. She received a 125 mg methylprednisolone intramuscular injection in the office and began 20 mg oral prednisone with a decreased dose schedule the following day. She presented to the dermatology office 3 days later with persistent symptoms.

Physical examination in the dermatology clinic revealed an erythematous morbilliform rash over the trunk (shown in Fig. 1), bilateral upper extremities, and lower extremities. Erythematous scaly plaques were present on the forearms and hands (shown in Fig. 1, 2). No oral or mucosal involvement was noted. Examination of the right and left upper extremities, hands, digits, and nails was otherwise normal.

**Fig. 1.**
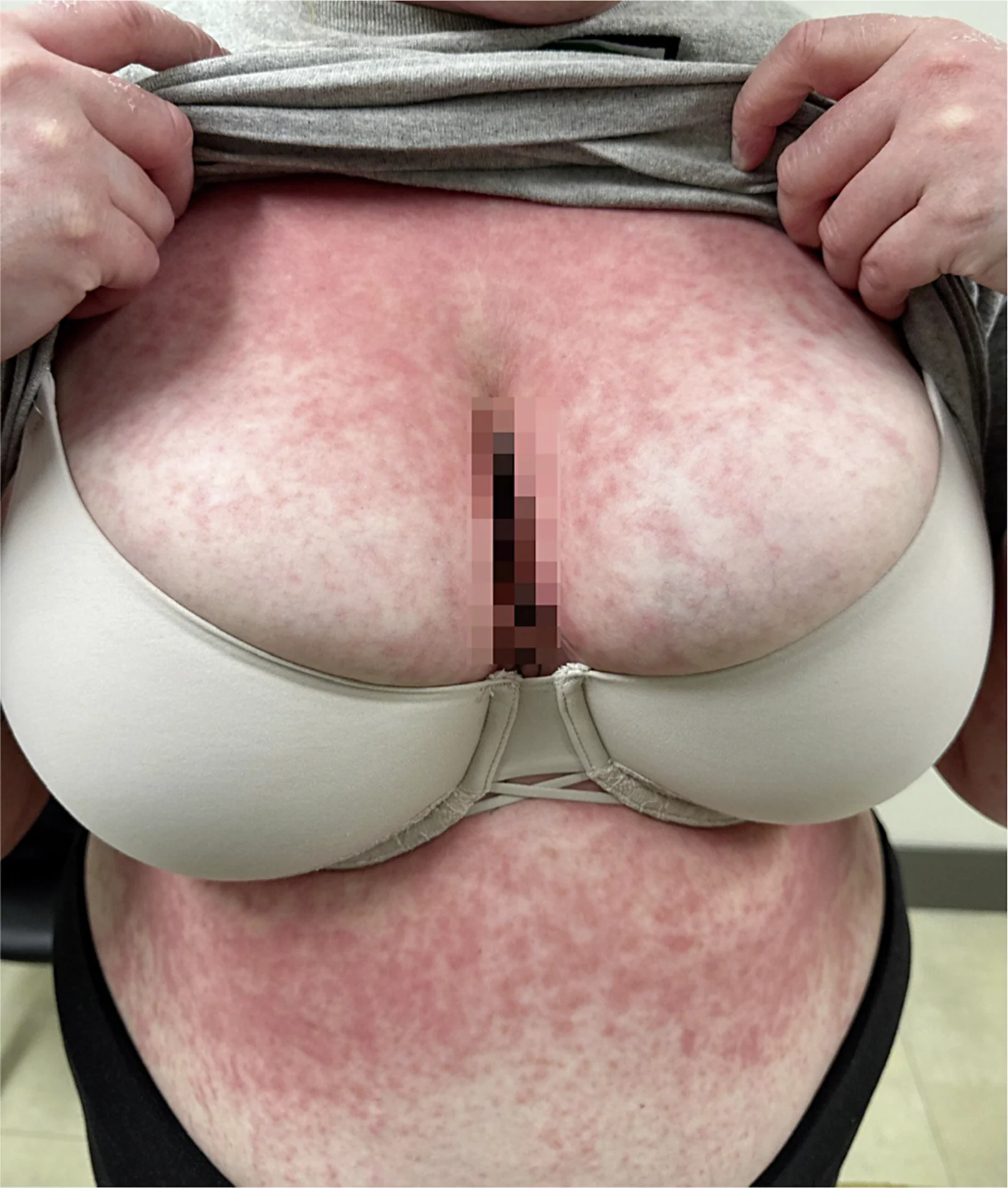
Erythematous morbilliform eruption on the chest and abdomen. Image edited to obscure patient-identifying features.

**Fig. 2.**
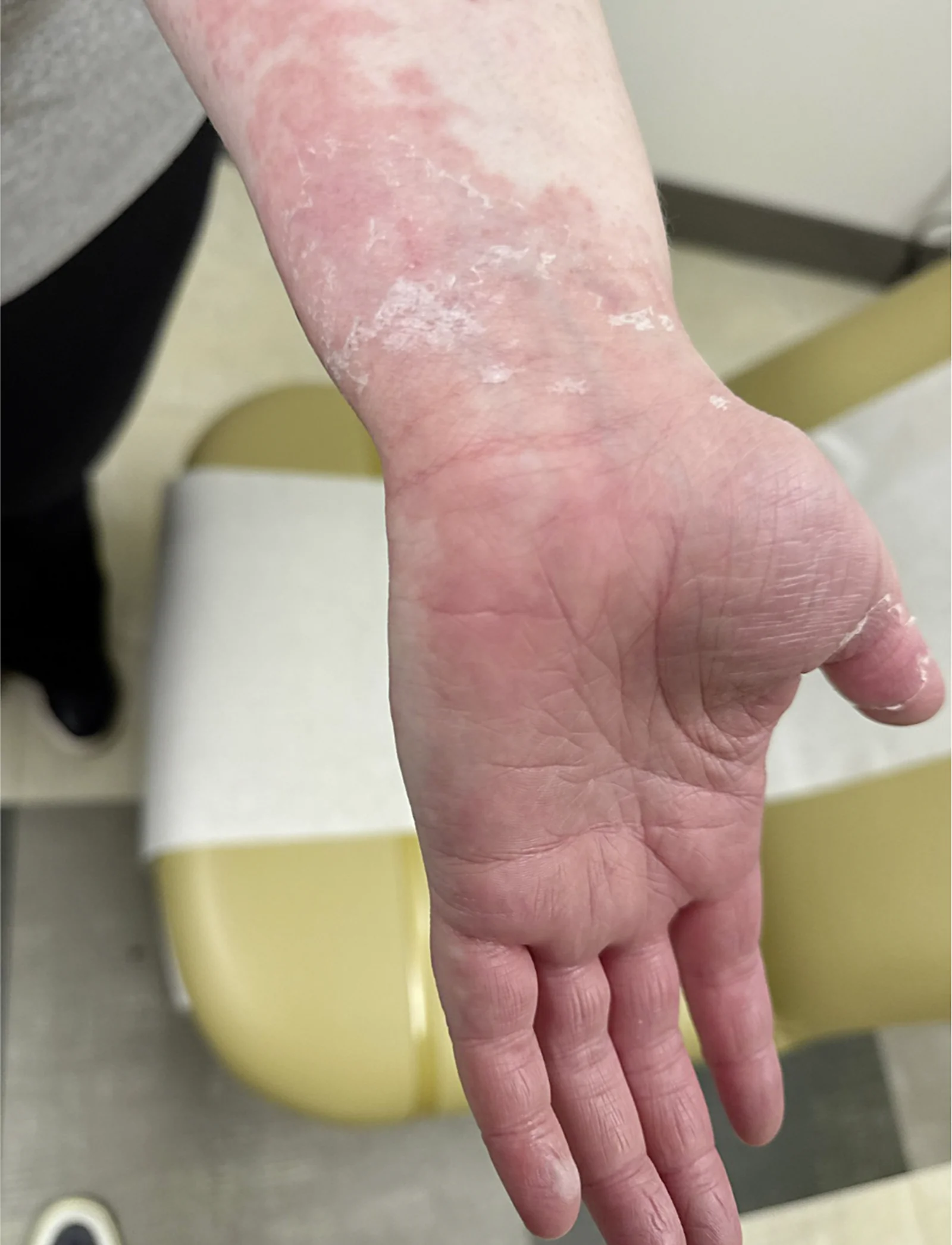
Erythematous scaly plaques on the left forearm and palm.

Complete blood cell count, comprehensive metabolic panel, and thyroid-stimulating hormone were obtained and were within normal limits. The patient returned the following day with persistent symptoms. A punch biopsy was performed on the right abdomen. Local anesthesia of 0.5% lidocaine was administered, and a 4 mm disposable trephine punch was used. Closure of the biopsy site was completed using a 4-0 nylon suture. Pathology results revealed histologic findings of large collections of intracorneal neutrophils overlying parakeratosis, confluent parakeratosis, a diminished but preserved granular layer, acanthosis, and focal spongiosis. Superficial perivascular lymphocytic infiltrate with neutrophils and extravasated erythrocytes was also reported with a lack of prominent eosinophils suggestive of psoriasiform features. The medication was discontinued and the eruption resolved within the following weeks. The patient was reevaluated in the dermatology clinic 1 year later and reported no recurrence of similar skin manifestations. The clinical presentation, histopathological features, timeline of drug association, and lack of similar skin findings in the future are suggestive of a psoriasiform drug eruption.

Differential diagnosis included plaque psoriasis, eczema, morbilliform drug eruption, lichenoid drug eruption, and acute generalized exanthematous pustulosis (AGEP). Plaque psoriasis and eczema are considered due to the sudden eruption of inflamed, erythematous, dry skin with diffuse spread; however, the resolution of skin symptoms with drug cessation is suggestive of a drug-induced reaction. Morbilliform drug reaction is difficult to confirm by skin biopsy, and histopathological findings can be nonspecific [3]. At the time of clinical evaluation, the patient’s cutaneous presentation resembled a morbilliform eruption; however, the biopsy results and poor response to corticosteroids were more consistent with a psoriasiform drug eruption. Although lichenoid drug reactions can present with pruritic eruptions, we would expect violaceous papules and plaques rather than an erythematous eruption. In this case, the use of systemic steroids before dermatological evaluation adds to the diagnostic complexity due to the possible alteration of clinical, histological, and laboratory findings from the anti-inflammatory effects of corticosteroids. AGEP typically presents with fever and a more rapid progression of symptoms along with cutaneous reactions in the intertriginous areas. The delayed presentation to dermatology means there may have been pustules during the initial phase of the eruption. At the time of evaluation, the scale morphology was not specifically suggestive of the collarette desquamation seen in resolving pustular drug eruption. Still, it is recognized that steroid use could have altered these findings. Although histopathologic overlap exists between AGEP and psoriasiform drug eruptions, the overall architectural pattern seen in the histology favors a psoriasiform drug eruption, characterized by confluent parakeratosis, acanthosis, diminished but preserved granular layer, intracorneal neutrophils, and lack of prominent eosinophils [4, 5]. In this case, the punch biopsy and histopathological findings were critical in gaining insight into the cutaneous morphology present in this patient; however, prior systemic corticosteroid use before clinical presentation creates diagnostic uncertainty and the differentials listed above cannot be excluded.

## Discussion

The development of a psoriasiform drug eruption following initiation of fremanezumab is a rare and poorly understood phenomenon. To date, few reports in the literature have described psoriasis triggered or unmasked by CGRP inhibitors. This case is notable for a psoriasiform drug eruption in a patient with no personal or family history of psoriasis, raising the possibility of a direct immunologic effect related to CGRP blockade.

### Diagnostic Challenge

On presentation to the dermatology clinic, the patient demonstrated an erythematous morbilliform eruption on the trunk and erythematous scaly plaques on the forearms and hands. The patient received both oral and intramuscular steroids before dermatologic evaluation, possibly modifying the surface appearance of the eruption, masking psoriasiform features, and reducing scale on the trunk. The mixed clinical and histopathologic features represent the importance of an appropriate medication history and biopsy in persistent cutaneous eruptions. The biopsy site was performed on the abdomen where the eruption appeared primarily morbilliform; however, the histopathology supported a psoriasiform drug eruption rather than a morbilliform drug reaction. Distinguishing psoriasiform drug eruption from new-onset psoriasis can be clinically challenging, particularly in patients without prior dermatologic history. In this case, the eruption began at the injection site in the abdomen and progressed diffusely, initially resembling a drug eruption, followed by improvement of symptoms after drug discontinuation. In an initial plaque psoriasis presentation, lesions are often localized and distributed along the extensor surfaces, such as the knees and elbows, with more prominent scaling [6]. The lack of improvement to systemic steroids and histopathology cannot reliably distinguish between a psoriasiform drug eruption and classic psoriasis due to significant overlap. The patient had complete resolution of symptoms with the discontinuation of the medication and no episodes of recurrence. These findings suggest a drug eruption rather than a coincidental first presentation of psoriasis; however, diagnostic uncertainty exists due to the absence of classic psoriasis features and nonspecific histopathology. Therefore, new-onset psoriasis cannot be excluded.

### Literature Context

A limited number of published cases have reported psoriatic flares associated with CGRP inhibitors. In a recent case series, two patients experienced worsening of pre-existing psoriasis or psoriatic arthritis shortly after initiation of CGRP monoclonal antibodies, with symptoms appearing after just one to two doses [2]. Unlike those reports, the present case represents a psoriasiform drug eruption in an individual with no underlying psoriatic disease, suggesting that CGRP inhibition may act not only as a flare trigger but also as a potential inciting factor in genetically or immunologically predisposed patients.

### Proposed Pathophysiology

Psoriasis is a chronic inflammatory cutaneous disease with multifactorial pathogenesis. The factors involved are behavioral, environmental, genetic, and immune-mediated processes. Immune cell dysregulation in the skin contributes to the skin manifestations present in individuals with psoriasis. The IL-23-driven immune axis promotes Th1 and Th17 responses. Activated Th17 cells produce IL-17 and IL-22, key effector cytokines involved in keratinocyte hyperproliferation and cutaneous inflammation [7]. CGRP is a neuropeptide that is widely expressed in sensory nerves and involved in neuroimmune modulation in the skin. It influences dendritic cell function, cytokine signaling, and skin homeostasis maintenance [8]. The loss of CGRP-mediated immune effects may lead to disruption of this immune balance, leading to a relative pro-inflammatory shift and providing a potential mechanism for psoriasiform inflammation.

## Conclusion

This case highlights the importance of considering immune-mediated inflammatory skin conditions in patients presenting with dermatological manifestations after the initiation of CGRP inhibitors. The association between psoriasiform drug eruption and CGRP inhibitors remains rare but represents a potential manifestation that clinicians should be made aware of as these drugs were not in clinical use until 2018. Early recognition of these dermatological manifestations is important and can allow early discontinuation of the medication if indicated.

## Statement of Ethics

This study was performed in accordance with the Declaration of Helsinki. This case report did not require ethical approval in accordance with local/national guidelines. Written informed consent was obtained from the individual for publication of the details of their medical case and any accompanying images.

## Conflict of Interest Statement

The authors have no conflicts of interest to declare.

## Funding Sources

This study was not supported by any sponsor or funder.

## Author Contributions

K.C.: contributed to the manuscript by gathering information for the introduction, literature review, drafting, reviewing, and editing the manuscript. The author agreed to the final version of the paper and accepted accountability for all aspects of the work. M.R. and E.S.: contributed to the manuscript by gathering and organizing information for the case report, literature review, drafting, reviewing, and editing the manuscript. The author agreed to the final version of the paper and accepted accountability for all aspects of the work. A.G., S.C., M.D., and P.A.-C.: contributed to the manuscript by gathering clinical information used in the case report, including patient history, images, manuscript drafting, and review. The author agreed to the final version of the paper and accepted accountability for all aspects of the work.

## Data Availability

All data generated or analyzed during this study are included in this article and its online supplementary material files. Further inquiries can be directed to the corresponding author.

## References

[1] Dodick DW , Silberstein SD, Bigal ME, Yeung PP, Goadsby PJ, Blankenbiller T, . Effect of fremanezumab compared with placebo for prevention of episodic migraine. JAMA. 2018;319(19):1999–2008. 10.1001/jama.2018.4853 PMC6583237 29800211

[2] Ray JC , Allen P, Bacsi A, Bosco JJ, Chen L, Eller M, . Inflammatory complications of CGRP monoclonal antibodies: a case series. J Headache Pain. 2021;22(1):121. 10.1186/s10194-021-01330-7 PMC8501661 34625019

[3] Yan A , Madigan L, Korman A, Shearer S, Dulmage B, Patel T, . Morbilliform eruptions: differentiating low-risk drug eruptions, severe cutaneous adverse reactions, viral eruptions, and acute graft-versus-host disease. Am J Clin Dermatol. 2025;26(3):379–93. 10.1007/s40257-025-00924-0 PMC12085335 39888589

[4] Isom J , Braswell DS, Siroy A, Auerbach J, Motaparthi K. Clinical and histopathologic features differentiating acute generalized exanthematous pustulosis and pustular psoriasis: a retrospective series. J Am Acad Dermatol. 2020;83(1):265–7. 10.1016/j.jaad.2020.03.015 32173359

[5] Parisi R , Shah H, Navarini AA, Muehleisen B, Ziv M, Shear NH, . Acute generalized exanthematous pustulosis: clinical features, differential diagnosis, and management. Am J Clin Dermatol. 2023;24(4):557–75. 10.1007/s40257-023-00779-3 PMC10166469 37156992

[6] Koca TT . A short summary of clinical types of psoriasis. North Clin Istanb. 2016;3(1):1–5. 10.14744/nci.2016.16023 PMC5175084 28058392

[7] Branisteanu D , Cojocaru C, Diaconu R, Porumb EA, Alexa AI, Nicolescu AC, . Update on the etiopathogenesis of psoriasis. Exp Ther Med. 2022;23(3):201. 10.3892/etm.2022.11124 PMC8794554 35126704

[8] Kim YJ , Granstein RD. Roles of calcitonin gene-related peptide in the skin, and other physiological and pathophysiological functions. Brain Behav Immun Health. 2021;18:100361. 10.1016/j.bbih.2021.100361 PMC8551410 34746878

